# Impact of Holding a Badminton Racket on Spatio-Temporal and Kinetic Parameters During Manual Wheelchair Propulsion

**DOI:** 10.3389/fspor.2022.862760

**Published:** 2022-06-27

**Authors:** Ilona Alberca, Félix Chénier, Marjolaine Astier, Marion Combet, Sadate Bakatchina, Florian Brassart, Jean-Marc Vallier, Didier Pradon, Bruno Watier, Arnaud Faupin

**Affiliations:** ^1^IAPS, Université de Toulon, La Garde, France; ^2^Mobility and Adaptive Sports Research Lab, Department of Physical Activity Sciences, Université du Québec à Montréal, Montreal, QC, Canada; ^3^Centre for Interdisciplinary Research in Rehabilitation of Greater Montreal, Institut Universitaire sur la Réadaptation en Déficience Physique de Montréal, Montreal, QC, Canada; ^4^Université de Toulon, LAMHESS, EA 6312, La Garde, France; ^5^Pole Parasport - ISPC Synergies, Hôpital Raymond-Poincaré, Garches, France; ^6^LAAS-CNRS, Université de Toulouse, CNRS, UPS, Toulouse, France

**Keywords:** biomechanics, wheelchair, risk of injury, propulsion effectiveness, Para badminton

## Abstract

**Introduction:**

Para badminton entered the Paralympic world for the first time with the 2021 Paralympic Games in Tokyo. The particularity of this sport lies in the handling of the wheelchair and the racket simultaneously. To the best of our knowledge, and considering the youthfulness of this sport, it appears that no study has looked at the impact of the badminton racket on the kinetic and spatiotemporal parameters. Therefore, the aim of our study was to investigate the impact of the badminton racket on the amplitude of kinetic and spatiotemporal parameters of wheelchair propulsion, considered as propulsion effectiveness and risk of injury criteria. We hypothesized that holding a badminton racket while propelling the wheelchair modifies the kinetics and temporal parameters of the athlete's propulsion due to the difficulty to hold the handrim, therefore decreasing propulsion effectiveness and increasing risk of injury.

**Materials and Methods:**

For six 90-min sessions, 16 able-bodied individuals were introduced to badminton. No injuries hindered their propulsion. They had to propel with and without a racket held on the dominant side along a 20 m straight line at a constant velocity of 5 km/h. They all used the same sports wheelchair equipped with two instrumented wheels (SmartWheel).

**Results:**

Participants increased their maximal total force and force rate of rise but decreased their fraction of effective force with their dominant hand compared to the non-dominant hand when using a racket. In addition, they decreased their fraction of effective force, push time, cycle time, and push angle, and increased their maximal propulsive moment, maximal total force, and force rate of rise when comparing the same dominant hand with and without the racket.

**Discussion:**

Using a badminton racket modifies the athlete's force application in a way that is generally related to lower propulsion effectiveness and a higher risk for injury. Indeed, it seems that propulsion with a racket prevents from correctly grabbing the handrim.

## Introduction

Para badminton is a young sport as it was first played in the 1990s when several German athletes became interested in adapting the rules of classical badminton for the people with disabilities. It entered the Paralympic world for the first time with the 2021 Paralympic Games in Tokyo.

Small-court wheelchair sports, such as Para badminton, are described as intermittent aerobic activities that are interspersed with brief periods of high-intensity work (Coutts, 1992; Bloxham et al., 2001; Goosey-Tolfrey et al., 2006; Roy et al., 2006; Mota and Almeida, 2020). The nature of the discipline requires athletes to perform rotations, abrupt forward and backward movements, and short sprints. The different shots performed by the players such as the release, the smash, or the drive require high-intensity efforts (Yüksel, 2018a,b). Like wheelchair tennis, the originality of this sport lies in the handling of the wheelchair while holding and using a racket. To the best of our knowledge, no study has investigated the impact of the badminton racket on propulsion effectiveness and risk of injury. However, the wheelchair tennis has been the subject of more studies, some of which focusing on the impact of the racket on kinetic and temporal parameters of the propulsion. These studies have shown that:

- Maximal velocity is reduced on the first three pushes with a racket (Goosey-Tolfrey and Moss, 2005).
- Power loss and power output generation are higher with the racket due to the longer time needed to couple the hand with the racket to the rim (de Groot et al., 2017).
- The arm holding the tennis racket has to withstand higher forces when propelling the wheelchair in sprints, compared to the arm without the racket (de Groot et al., 2017).

Taken together, these findings in wheelchair tennis suggest that the use of the racket induces adjustment in the mechanical spatiotemporal parameters of the athletes related to a decrease in propulsion effectiveness (Goosey-Tolfrey and Moss, 2005; de Groot et al., 2017). Likewise, an increase in the forces carried by an upper limb is associated with an increased risk of injury (Boninger et al., 2005).

The area of interest here is Para badminton, which remains largely unstudied in the scientific literature. However, wheelchair tennis and Para badminton are being the two disciplines close to each other; we can assume that in badminton also, the racket could have a negative impact on the propulsion effectiveness and the injury risk of the athletes. Propulsion effectiveness and injury risk are related to several kinetic and spatio-temporal parameters such as total force, propulsive moment, force rate of rise, fraction of effective force, power, push and cycle time, and push angle (Boninger et al., 2000, 2005; Chow et al., 2001; de Groot et al., 2002, 2008; Goosey-Tolfrey and Moss, 2005; Koopman et al., 2016). Comprehensive analysis including forces developed by the athletes would allow calculating parameters related to the propulsion effectiveness and the risk of injury. Therefore, the aim of our study is to investigate the impact of holding a badminton racket on the kinetic and spatiotemporal parameters of wheelchair propulsion. Specifically, we would like to analyze the impact of the badminton racket during wheelchair propulsion on maximal total force, maximal propulsive moment, rate of rise, fraction of effective force, maximal power output, push and cycle time and push angle. Those are essential parameters that can impact propulsion effectiveness, defined here as the ability to reach and maintain a given velocity, and risk of injury. Based on results in wheelchair tennis we hypothesized that wheelchair propulsion while holding a badminton racket modifies the kinetics and temporal parameters of the athlete's propulsion due to the difficulty to hold the handrim, therefore decreasing propulsion effectiveness and increasing risk of injury (Goosey-Tolfrey and Moss, 2005; Sindall et al., 2013; de Groot et al., 2017).

## Materials and Methods

### Study Design

The design of our study focused on the comparison of the measured parameters according to two conditions: propulsion without holding a badminton racket and propulsion while holding a badminton racket. In order to make this comparison and after a 5-min wheelchair warm-up, participants had to propel along with a 20-meter straight line at a constant velocity of 1.4 m/s (5 km/h) using a regular sound signal in a sports complex. They started the test at a standstill. Markers were placed at regular intervals along the 20-meter straight line. Each time the signal sounded; the participant had to be at the next markers, and so on for each marker until the end of the 20 meters. The participant had to propel continuously without braking or accelerating abruptly. To get used to the sound system, the participants were allowed to practice the course prior to the registration of the trial. Two passages were made in a randomized order: with and without a badminton racket. The racket was the same for all participants (Yonex Astrox Smash Navy Blue, 73 g) and was held on the dominant side. Because the test was submaximal, a 1-min recovery time was implemented between each trial.

### Setting

The tests done in this study were performed at the University of Toulon (La Garde, France) on November 21, 2018. The experimental protocol was approved by the Comité d'Ethique pour les Recherches en STAPS (CERSTAPS) from Conseil National des Universités de France [certificate #CERSTAPS 2018-16-07-26] filed on June 6, 2018 and accepted on July 7, 2018. Participants were recruited starting in September, 2018.

### Participants

Our study included 16 able-bodied sports students. Our exclusion criteria were injury or pain that could interfere with wheelchair propulsion. We used a statistical power test to determine the sample size needed for the study. The article by de Groot et al. (2017) was used as a reference for this test. Thus, for a statistical power of 0.95, the calculation of statistical power gave us an average of 8 participants for the statistical tests we wished to perform on our measures. Based on this average, 16 participants were included in the study. Statistical power was calculated using G^*^Power software (G^*^ Power, 2020; g-power.apponic.com). All participants were introduced to wheelchair maneuverability and Para badminton during 6 practice sessions of 90 min. They were novices in wheelchair handling and wheelchair propulsion. These practice sessions are part of their school curriculum in Sciences et Techniques des Activités Physiques et Sportives (STAPS). Characteristics of all participants are presented in Table 1.

**Table 1:** Participants' characteristics.

**Participant**	**Gender**	**Age (years)**	**Height (cm)**	**Body mass (kg)**	**BMI (kg/m^**2**^)**	**Dominant hand**
S1	Man	42	180	75	23.2	R
S2	Man	27	179	65	20.3	R
S3	Woman	20	165	60	22.0	R
S4	Man	22	175	95	31.0	R
S5	Man	21	180	75	23.2	R
S6	Man	21	179	75	23.4	R
S7	Man	21	171	64	21.9	R
S8	Man	20	174	61	20.2	R
S9	Woman	21	169	52	18.2	R
S10	Woman	24	172	59	19.9	L
S11	Woman	19	161	50	19.3	R
S12	Man	19	176	77	24.9	L
S13	Woman	20	170	63	21.8	R
S14	Woman	22	163	62	23.3	L
S15	Man	19	175	95	31.0	R
S16	Man	22	175	63	20.6	R
Mean(SD)		22.5(5.6)	172.8(5.9)	68.2(13.1)	22.8(3.7)	

*With SD, standard deviation; BMI, Body Mass Index*.

### Data Measurement

Participants used a single multi-sport wheelchair with a wheel size of 26 inches and a camber angle of 18 degrees, which is similar to chairs used in Para badminton. The chair was equipped bilaterally with two instrumented wheels (SMARTWheel. 2013 edition, Outfront LCC). Measurement tools such as instrumented wheels allow to measure parametersin conditions close to the original discipline and without impeding propulsion. These wheels have a weight and moment of inertia of ~4.9 and 0.15 kg·m^2^ (Sprigle et al., 2016). With these tools, we can measure the wheel angle θ, forces *F*_*x*_, *F*_*y*_, *F*_*z*_(F_y_ is the force applied up and down on the pushrim; F_x_ is force applied laterally; F_z_ is the force out of the plane of the wheel *SmartWheel 2008*^1^ p. 46. *Users Guide*, 2014) and force moments *M*_*x*_, *M*_*y*_, *M*_*z*_applied on each handrim for all sessions at 240 Hz. Dynamic kinetic offsets were canceled using a method described in Chénier et al. (2017) because the recorded kinetics may include dynamic offsets that affect the accuracy of the measurements. Wheelchair velocity was calculated from wheel angles using a 131-point first-order Savitzky-Golay derivative filter (Chénier et al., 2015).

All pushes recorded by the instrumented wheels were segmented. A 30 N threshold selected experimentally based on the recorded dataset helped us to make this segmentation. This automated segmentation was manually checked for each of the push for each trial to correct any errors. For each run, the first two and last pushes were excluded and considered as transitional pushes.

### Variables

The parameters presented in Table 2 were calculated and averaged over all the selected pushes in a bilateral manner. Thus, we obtained kinetic and spatiotemporal data for the dominant and non-dominant hand of each participant.

**Table 2:** Description and equations for the outcome measures.

**Parameters**	**Description**	**Equations**
**Pushrim kinetics**		
Maximal total force (Ftot_peak_) [N]	Sum of the maximal forces in the 3 planes of space applied to the handrim for each push	*max*(*sqrt*(*Fx*^2^+*Fy*^2^+*Fz*^2^))
Maximal propulsive moment (Mz_peak_) [Nm]	Maximal propelling moment applied to the handrim for each push	Calculation carried out by the SmartWheel software
Rate of rise (RoR) [N.s^−1^]	Rate of rise in maximal total force for each push	$\frac{dFtot_{\max}}{dt}$
Fraction of Effective Force (FEF) [%]	Percentage of forces useful for propulsion	$abs\left(\frac{Ftan}{Ftot}\right)x\;100$
Maximal power output (PO_peak_) [W]	Maximal power output developed by the participant to the handrim for each push	*peak*[θ × *Mz*]
Angular impulse (AI) [Nm.s]	Gain of propulsive moment during one push	*Mz*_*mean*_ *x PT*
**Temporal parameters**		
Push time (PT) [s]	Contact time between hand and wheelchair handrim	*t*_*end*_(*i*)− *t*_*start*_(*i*)
Cycle time (CT) [s]	Time between the start of first push and next push for each push	*t*_*start*2_(*i*)−*t*_*start*1_(*i*)
Push angle (PA) [°]	Wheel angle course during push time	Calculation carried out by the SmartWheel software

*With Fx, horizontal force; Fy, vertical force; Fz, mediolateral force; r, wheel radius; start, start of a push; end, end of a push; t, time (s); v, wheel velocity; i, push considered*.

All data processing and calculations were performed using Python/SciPy and the Kinetics Toolkit library (Chénier, 2021).

### Statistical Methods

A total of 10 variables were calculated. The means and standard deviation of these variables were calculated per condition and per limb separately. All data were analyzed using SPPS version 20 (SPSS Inc., Chicago, Illinois USA).

The Shapiro–Wilk test showed that all outcomes' measures were not normally distributed. Thus, the statistical analyses were performed on the log-transformed data. A repeated measures ANOVA was then performed (with two within factors: with racket vs. without racket; dominant vs. non-dominant hand) to look at the existing differences between dominant and non-dominant hand according to the with-or-without-racket condition. A Mauchly sphericity test was performed to check if the sphericity hypothesis was violated. This was the case for all the calculated variables. A Greenhouse-Geisser correction was applied. A Bonferroni adjustment was made for multiple comparisons with *p* = 0.05. For each significant difference, the effect size $\eta_{p}^{2}$ was calculated using the following equation:


(1) $$\begin{array}{r} \eta_{p}^{2}=\;\frac{SS_{effect}}{SS_{effect}+\;SS_{error}} \end{array}$$

*With* $\eta_{p}^{2}$*: partial eta-squared of the considered variable;* *SS*_*effect*_*: effect sums of squares of the considered variable;* *SS*_*error*_*: error sums of squares of the considered variable*.

Effect size was interpreted according to Cohen (1988): small ($\eta_{p}^{2}$ = 0.01), medium ($\eta_{p}^{2}$ = 0.06), and large ($\eta_{p}^{2}$ = 0.14).

We also performed a paired student test to compare the parameters of the same hand with and without a racket on the log-transformed data. Statistical significance was set at *p* < 0.05. For each significant difference, the effect size d was calculated using the following equation:


(2) $$d=\;\frac{mean(X_{0})-mean(X_{1})}{s.d.(X_{0})}$$

*With X: studied parameter, 0: data without racket or dominant hand according to the statistical analysis and 1: data with racket or non-dominant hand according to the statistical analysis*.

Effect size was interpreted according to (Cohen, 1988): small (*d* = 0.2), moderate (*d* = 0.5), and large (*d* = 0.8) (Cohen, 1988).

## Results

We checked the average velocity of the participants to ensure that the constant velocity requirement was met. The participants reached a mean velocity of 1.44 m/s during the runs with racket and 1.42 m/s during the runs without racket, which corresponds to the imposed velocity.

### Bilateral Analysis

The results of the bilateral analysis are presented in Table 3. When comparing the results of both hands with and without a racket, an effect of the racket was found for all parameters except AI. Indeed, with racket P_peak_ (*p* = 0.028) and Mz_peak_ (*p* = 0.009) increase slightly and Ftot_peak_ (*p* < 0.001) and Ror (*p* < 0.001) increase largely. On the contrary, FEF (*p* < 0.001) decreases largely, PA (*p* = 0.001) decreases slightly and PT (*p* < 0.001) and CT (*p* < 0.001) decrease moderately in condition with racket. Significant differences between dominant and non-dominant hand regardless of the condition were noted for P_peak_ (*p* = 0.013), Mz_peak_ (*p* = 0.018), Ftot_peak_ (*p* < 0.001), FEF (*p* < 0.001), and Ror (*p* < 0.001). P_peak_ and Mz_peak_ are slightly higher on the dominant hand and Ftot_peak_ and Ror are largely higher on the dominant side. Conversely, FEF is largely lower on the dominant side compared to the non-dominant side. Finally, an interaction between the condition and the side considered was found for Ftot_peak_ (*p* < 0.001) and FEF (*p* < 0.001).

**Table 3:** Kinetic and spatiotemporal parameters in 20-meter wheelchair straight propulsion according to the condition (with racket, without racket) and upper limb (dominant, non-dominant).

	**With racket**			**Without racket**			**ANOVA**								
	**D**	**ND**		**D**	**ND**		**Condition effect**			**Side effect**			**Interaction (Condition** **×Side)**		
	**Mean (SD)**	**Mean (SD)**	**D**	**Mean (SD)**	**Mean (SD)**	**d**	***F***	***p***	**η^2^**	***F***	***p***	**η^2^**	***F***	***p***	**η^2^**
P_peak_ [W]	112.53 (63.74)	105.00 (51.43)	0.130	104.78 (65.87)	95.98 (55.43)	0.145	**4.879**	**0.028**	**0.025**	**6.298**	**0.013**	**0.032**	0.252	0.616	0.001
Mz_peak_ [N.s]	22.24 (8.21)	21.61 (8.76)	0.074	20.55 (10.53)	19.56 (10.22)	0.095	**7.049**	**0.009**	**0.036**	**5.680**	**0.018**	**0.029**	0.343	0.559	0.002
Ftot_peak_ [N]	**117.77 (45.36)**	86.53 (40.75)*	**0.725**	**86.53 (38.65)**	73.75 (38.81)*	**0.330**	**32.738**	**<0.001**	**0.148**	**123.513**	**<0.001**	**0.395**	**12.211**	**<0.001**	**0.061**
FEF [%]	**29.36 (6.93)**	40.85 (14.15)*	**1,031**	**35.82 (10.94)**	41.14 (13.14)*	**0.440**	**33.888**	**<0.001**	**0.152**	**124.709**	**<0.001**	**0.398**	**25.260**	**<0.001**	**0.118**
Ror [N/s]	587.42 (305.96)	388.64 (228.52)	0.736	388.65 (210.90)	289.02 (181.49)	0.506	**43.815**	**<0.001**	**0.188**	**118.824**	**<0.001**	**0.386**	1.765	0.186	0.009
AI [Nm.s]	4.01 (2.00)	4.00 (2.19)	0.005	4.25 (2.41)	4.10 (2.44)	0.062	0.102	0.750	0.001	0.286	0.594	0.002	1.191	0.276	0.006
PT [s]	0.34 (0.10)	0.34 (0.08)	0	0.36 (0.07)	0.37 (0.07)	0.143	**12.254**	**<0.001**	**0.061**	0.003	0.955	0.000	0.004	0.950	0.000
CT [s]	1.13 (0.43)	1.11 (0.39)	0.049	1.29 (0.45)	1.30 (0.45)	0.022	**12.797**	**<0.001**	**0.063**	0.394	0.531	0.002	0.685	0.409	0.004
PA [°]	84.68 (30.47)	83.22 (19.44)	0.057	90.98 (19.60)	91.06 (19.23)	0.004	**10.886**	**0.001**	**0.054**	0.196	0.659	0.001	0.043	0.836	0.000

*With racket, racket held in the dominant hand; D, dominant hand; ND, non-dominant hand; Condition, with or without racket; Side, dominant or non-dominant hand; SD, standard deviation*.

^*^Significant difference in Post hoc pair wise comparisons with Bonferroni adjustment (dominant vs. non-dominant hand) with p < 0.001.

*F, result of the ANOVA; p, p-value fixed at 0.05; d, effect size for the significant difference in Post hoc pair wise comparisons with Bonferroni adjustment (dominant vs. non-dominant hand); η^2^, effect size for the significant difference in the ANOVA. Bold values indicate the significant values*.

### Unilateral Analysis

The results of the unilateral analysis of the data are presented in Table 4. When we compare the same dominant hand with and without racket, we note that P_peak_ (*p* = 0.032) and Mz_peak_ (*p* = 0.010) are slightly higher and Ftot_peak_ (*p* < 0.001) and Ror (*p* < 0.001) are largely higher with racket. While FEF (*p* < 0.001) is largely lower and PT (*p* = 0.001), CT (*p* < 0.001), and PA (*p* = 0.006) are slightly lower with racket compared to the passage without racket.

**Table 4:** Kinetic and spatiotemporal parameters in a 20-meter wheelchair straight propulsion of the same dominant hand with and without racket.

	**Dominant hand**		***T*** **-test**		
	**With racket**	**Without racket**	**With racket** **×Without racket**		
	**Mean (SD)**	**Mean (SD)**	**t**	***P***	**d**
P_peak_ [W]	112.53 (63.74)	104.78 (65.87)	**1.867**	**0.032**	**0.120**
Mz_peak_ [N.s]	22.24 (8.21)	20.55 (10.53)	**2.356**	**0.010**	**0.179**
Ftot_peak_ [N]	117.77 (45.36)	86.53 (38.65)	**7.530**	**<0.001**	**0.741**
FEF [%]	29.36 (6.93)	35.82 (10.94)	**8.197**	**<0.001**	**0.705**
Ror [N/s]	587.42 (305.96)	388.65 (210.90)	**7.597**	**<0.001**	**0.756**
AI [Nm.s]	4.01 (2.00)	4.25 (2.41)	0.330	0.371	0.108
PT [s]	0.34 (0.10)	0.36 (0.07)	**3.086**	**0.001**	**0.231**
CT [s]	1.13 (0.43)	1.29 (0.45)	**3.134**	**<0.001**	**0.363**
PA [°]	84.68 (30.47)	90.98 (19.60)	**2.555**	**0.006**	**0.246**

*With racket, racket held in the dominant hand; SD, standard deviation; t, results of the t-test; d, effect size for the significant difference; p, p-value fixed at 0.05; ANOVA. Bold values indicate the significant values*.

## Discussion

The design analyzing the impact of holding a badminton racket conducted in this article is, to our knowledge, the first of his kind in Para badminton. The objective of this article was to study the impact of the badminton racket on the amplitude of kinetic and spatiotemporal parameters of wheelchair propulsion. We hypothesized that wheelchair propulsion while holding a badminton racket modifies the kinetics of the athlete's propulsion. This hypothesis has been verified. Indeed, the use of the racket induces a negative impact on propulsion effectiveness when comparing the same hand with and without racket (fraction of effective force, push time, and push angle) and the dominant hand with racket vs. non-dominant hand (fraction of effective force). Although athletes can maintain the imposed constant overall velocity, their propulsion effectiveness is impacted. However, we must mention that only one propulsion effectiveness parameter (fraction of effective force) is impacted by the racket in the bilateral analysis of the data and that maximal propulsive moment increases slightly in the dominant hand with the racket compared to the same hand without the racket, which is positively related to better propulsion effectiveness. Moreover, the use of a badminton racket also seems to increase parameters related to risk of injury when comparing the dominant and non-dominant hand (maximal total force and rate of rise) and the same hand with and without the racket (maximal total force, rate of rise increased, and cycle time).

The increase in the maximal propulsive moment in the dominant hand during racket propulsion is accompanied by a moderate decrease in the fraction of the effective force, the push time, and the push angle. These parameters are related to propulsion effectiveness and our results appear to be consistent with a decrease in participant propulsion effectiveness. It is possible that the difficulty to grab the handrim of the wheelchair with the racket explains these results. Indeed, participant weakly increases their propulsive moment with the racket but with less continuity as evidenced by the push time and the push angle. Therefore, the proportion of forces that is useful for propulsion decreases. It seems that the wheelchair user makes shorter and reduced movements. For push time, de Groot et al. (2017) also looked at it in tennis and their study showed a decrease in push time and push angle, or contact angle as it is written in their study, with a tennis racket. These results are like ours although we do not deal with the same adapted sport. The decreases observed for these two parameters in the study of de Groot et al. (2017), are greater than those of our study. Indeed, the push time and the push angle decrease, respectively, by 18 and 20% in the study of de Groot et al. (2017) while in our study they decrease only by 5 and 8%. These differences may be due to the properties of the rackets. Indeed, a tennis racket is heavier and has a wider handle than a badminton racket. As a result, we can assume that the impact of a tennis racket is greater than that of a badminton racket. Moreover, we must remember that our study was carried out on able-bodied players. They therefore benefit from abdominal capacities that may be absent in people with disabilities. In addition, they have fewer skills than the Para badminton players.

The use of the racket appears to cause an increase in maximal total force when we look at the results of both hand with and without racket and the same dominant hand with and without a racket, resulting in a moderate increase in the rate of rise in the hand carrying the racket. It is possible to assume that the use of the racket hinders participants and prevents them from properly catching the handrim. They will then compensate for this lack of grip by applying more force on the handrim. In addition, the cycle time decreases when using the racket. For the same propulsion velocity, the participant made more and faster pushes, therefore increasing propulsion frequency. These sets of changes are considered to be risk factors for injury (Boninger et al., 2005). This result may be of particular interest for the coaches. Indeed, knowing that the use of the racket can increase the risk of injury, coaches can propose adapted sessions such as longer rest periods or specific active recoveries.

The results of the ANOVA show the existence of significant differences between dominant and non-dominant hand regardless of the propulsion condition. P_peak_ and Mz_peak_ are slightly higher on the dominant hand and Ftot_peak_ and Ror are largely higher on the dominant side. FEF is largely lower on the dominant side compared to the non-dominant side. These differences indicate the existence of an asymmetry between dominant and non-dominant hand for these parameters. Indeed, it seems that participants apply greater forces and powers on the dominant side than on the non-dominant side. These sets of changes are considered to be risk factors for injury (Boninger et al., 2005). Similarly, they appear to slightly produce more force useful for propulsion on the dominant side without this increasing their FEF. This indicates that the participant increases more forces not useful for the propulsion of the wheelchair, which is related to less propulsive effectiveness. It is possible that the participants' sport practice besides the study induced this asymmetry. Indeed, it is the case of asymmetrical sports practices such as racket sports that develop more muscle strength on the side of the limb carrying the racket. Several authors have also shown that one arm is specialized in a task compared to the second arm (Bagesteiro and Sainburg, 2002, 2003; Sainburg and Wang, 2002; Wang and Sainburg, 2003, 2004; Haaland, 2004; Sainburg and Schaefer, 2004; Schaefer et al., 2007). It is possible that the dominant limb is specialized in force production, unlike the non-dominant limb, which would explain this asymmetry.

We believe that the main limitation of this study concerns the group of participants. Indeed, our experiment was conducted on a population of able-bodied participants not experienced in wheelchair propulsion. The study on able-bodied participants provides homogeneous groups (Rice et al., 2010). However, for people who use manual wheelchairs daily, such as individuals with a paraplegia or tetraplegia, abdominal and trunk capabilities may be reduced due to the severity of the disability. Moreover, even though the participants were trained in Para badminton, badminton players have better racket handling technique than not experienced able bodied participants. The propulsion technique will differ from a novice participant to an expert in Para badminton. This influences propulsion, therefore inducing that our results will not be completely transferable to a population of people with disabilities. In addition to this limitation, we also studied wheelchair propulsion at constant velocity with and without a racket. However, this discipline mainly requires players to perform short sprints forward and backward. Our study being one of the first to look at the impact of the badminton racket on propulsion, we chose to carry out the tests in submaximal condition. This allows us to make a general assessment before being able to study the impact of the badminton racket in various conditions, to be sure that our results are the consequence of the addition of a condition (here the racket). Finally, the use of instrumented wheels increased the weight of the wheels, which may increase the rolling resistance of the wheelchair and its inertia. However, we believe that our results remain valid since the measurements are taken under the same conditions: we use two instrumented wheels that increase the rolling resistance in the same way on each side.

The objective of this study was to analyze the impact of the badminton racket on the kinetic parameters of wheelchair propulsion. We have highlighted that its use agrees with a modification of the kinetics of the participants related to a decrease of the propulsive effectiveness and an increase of the risks of injuries. To complete this analysis and to better understand the impact of the racket, future studies should be conducted under conditions encountered in playing Para badminton, such as consecutive forward and backward propulsion tests that approximate the movements encountered during practice. Moreover, an interesting aspect would also be to work on the comparison of the different possibilities of holding the badminton racket during propulsion. In the field of Para tennis, Koopman et al. (2016), have already been interested in testing different racket holding techniques. We could do the same in the field of Para badminton to complete the analysis of the impact of the racket on propulsion. Finally, proposing new handrim designs could be a solution to the difficulties encountered during propulsion with a badminton racket.

## Data Availability Statement

The original contributions presented in the study are included in the article/supplementary materials, further inquiries can be directed to the corresponding authors.

## Ethics Statement

The studies involving human participants were reviewed and approved by Comité d'Ethique pour la Recherche en Sciences et Techniques des Activités Physiques et Sportives. The patients/participants provided their written informed consent to participate in this study. Written informed consent was obtained from the individual(s) for the publication of any potentially identifiable images or data included in this article.

## Author Contributions

IA: data curation, formal analysis, and writing—original draft. FC: data curation and writing—review and editing. MA and MC: funding acquisition and methodology. SB and FB: funding acquisition and writing—review and editing. J-MV: project administration and validation. DP: data curation. AF: methodology, project administration, and writing—review and editing. All authors contributed to the article and approved the submitted version.

## Conflict of Interest

The authors declare that the research was conducted in the absence of any commercial or financial relationships that could be construed as a potential conflict of interest.

## Publisher's Note

All claims expressed in this article are solely those of the authors and do not necessarily represent those of their affiliated organizations, or those of the publisher, the editors and the reviewers. Any product that may be evaluated in this article, or claim that may be made by its manufacturer, is not guaranteed or endorsed by the publisher.

## Footnotes

^1^SmartWheel 2008 Users Guide (2014).
